# Curtailing patient-specific IMRT QA procedures from 2D dose error distribution

**DOI:** 10.1093/jrr/rrv084

**Published:** 2016-06-21

**Authors:** Keita Kurosu, Iori Sumida, Hirokazu Mizuno, Yuki Otani, Michio Oda, Fumiaki Isohashi, Yuji Seo, Osamu Suzuki, Kazuhiko Ogawa

**Affiliations:** 1 Department of Radiation Oncology, Osaka University Graduate School of Medicine, Osaka, 565-0871, Japan; 2 Department of Radiology, Osaka University Hospital, Osaka, 565-0871, Japan

**Keywords:** QA, IMRT, gamma index, prediction, MapCHECK

## Abstract

A patient-specific quality assurance (QA) test is conducted to verify the accuracy of dose delivery. It generally consists of three verification processes: the absolute point dose difference, the planar dose differences at each gantry angle, and the planar dose differences by 3D composite irradiation. However, this imposes a substantial workload on medical physicists. The objective of this study was to determine whether our novel method that predicts the 3D delivered dose allows certain patient-specific IMRT QAs to be curtailed. The object was IMRT QA for the pelvic region with regard to point dose and composite planar dose differences. We compared measured doses, doses calculated in the treatment planning system, and doses predicted by in-house software. The 3D predicted dose was reconstructed from the per-field measurement by incorporating the relative dose error distribution into the original dose grid of each beam. All point dose differences between the measured and the calculated dose were within ±3%, whereas 93.3% of them between the predicted and the calculated dose were within ±3%. As for planar dose differences, the gamma passing rates between the calculated and the predicted dose were higher than those between the calculated and the measured dose. Comparison and statistical analysis revealed a correlation between the predicted and the measured dose with regard to both point dose and planar dose differences. We concluded that the prediction-based approach is an accurate substitute for the conventional measurement-based approach in IMRT QA for the pelvic region. Our novel approach will help medical physicists save time on IMRT QA.

## INTRODUCTION

Intensity-modulated radiation therapy (IMRT) enables complex dose distribution around a target with minimized damage to normal tissues [1]. It requires multiple fluctuating beam intensities to conform the dose to targets; therefore, a patient-specific quality assurance (QA) test is conducted to verify the accuracy of dose delivery. The American Association of Physicists in Medicine (AAPM) Task Group 119 recommended that point dose measurement and planar dose verification would provide confidence for IMRT commissioning [2]. The criteria for machinery commissioning have also been applied to patient-specific IMRT QA, because optimized criteria that correlate to the patient dose–volume histogram (DVH) metric have not been established [3–5].

IMRT dose delivery often accompanies dynamic/static segment movement during the treatment; therefore, dosimetry tools are limited to having integrating dosimetric techniques. An ionization chamber is used for absolute point dose comparison between the measured and the calculated doses in the treatment planning system (TPS) [6]. Film verifies the planar dose by 3D composite irradiation with the same configuration in clinical treatments. Commonly, per-field measurement verifies the dose distribution on the coronal plane at a gantry angle of 0° using 2D diode array detectors or film. Patient-specific IMRT QA thus imposes a significant workload on medical physicists. Van Esch *et al*. investigated the time used by medical physicists for IMRT QA [7]. The time required to ascertain point dose difference and planar dose differences was 3–10 h per patient.

Pulliam *et al*. recently reported the results of patient-specific QA for 13 000 patients who received IMRT in their institution, suggesting the majority of point dose differences were within ±3% of tolerance, and failed plans were only a few percent outside tolerance [8]. Furthermore, the failure rate of gamma evaluation was ∼0.1% with regard to planar dose differences. The criterion for starting IMRT in their institution was 90% of the pixels passing the criterion of 5%(global)/3 mm without a lower dose threshold. Consequently, the passing rate for point dose difference was 97.7%, and that for planar dose differences was 99.3%. Therefore, while it is essential to verify the IMRT plan for every patient, the cost effectiveness of current patient-specific IMRT QA may be questioned.

We previously developed 3D dose prediction software that incorporates the 2D dose error distribution into the 3D dose calculation [9, 10]. Our novel algorithm does not require recalculation on the planned dose. Instead, our algorithm reflects the 2D relative dose error in the coronal plane to a correlated dose grid in each gantry angle. In contrast, the 3DVH software (Sun Nuclear Corp., Melbourne, FL, USA) uses a planned dose perturbation algorithm to evaluate the 3D delivered dose [11]. The difference between the two algorithms is in where the 2D relative dose error will feed back. The prediction accuracies of these algorithms have been reported elsewhere [5, 9, 12]. Olch validated the prediction accuracies for patient-specific IMRT QA by 3DVH software with regard to point dose difference and planar dose differences [13]. The results indicated that certain IMRT QA measurement procedures could be replaced by using 3DVH software with a constraint of 1% under-reading for the diode array detectors; however, the target IMRT location varied over a wide range. Pulliam *et al*. reported that notable variations with regard to point dose and planar dose differences were observed in particular in tumors of small size [8].

A 3D delivered dose prediction from per-field measurement allows the curtailing of QA procedures; however, a limited range of target sizes and locations should be evaluated for the first step. The objective of this study was, therefore, to reconsider the effectiveness of our 3D delivered dose prediction method in curtailing IMRT QA procedures for large targets.

## MATERIALS AND METHODS

### Patients and IMRT planning

The object of this study was IMRT QA for the pelvic region, for which dose differences were within a certain range in the study of Puliam *et al*. [8]. A seven-field coplanar treatment plan with beam angles of 27°, 78°, 129°, 180°, 231°, 282° and 333° was generated with a 6-MV flattened or an 11-MV unflattened X-ray beam using an ARTISTE^TM^ accelerator with 160 multileaf collimator (MLC) (Siemens, Erlangen, Germany). The beam property of the ARTISTE^TM^ is described elsewhere [14]. Fifteen patients who had undergone IMRT for uterine cancer in our institution between February 2014 and December 2014 were enrolled in this study. A radiation oncologist delineated the contouring of the clinical target volume as the target, and then a medical physicist created the IMRT plan. A mean dose of 50.4 Gy was prescribed for the planning target volume (PTV) over 28 fractions for all patients [15]. Treatment plans were created using the XiO TPS (Elekta, Stockholm, Sweden), and a dose calculation algorithm was a superposition with a voxel size of 2.0 × 2.0 × 2.0 mm^3^.

### Conventional IMRT QA

**Fig. 1.**
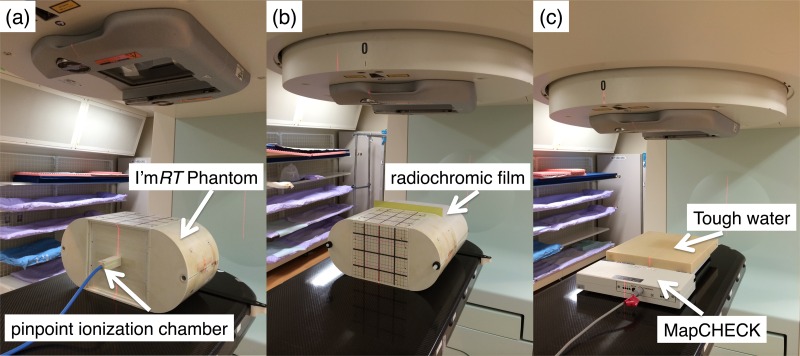
Schematic layouts of our conventional IMRT QA are shown. (**a**) The absolute point dose was measured using a PinPoint ionization chamber, which was inserted into the I'm*RT* Phantom. (**b**) The composite planar dose was measured using a GAFCHROMIC EBT3 film, which was also inserted into the phantom in the axial plane. (**c**) Per-field coronal planar dose was measured at a gantry angle of 0° using a MapCHECK device with Tough water of 80-mm thickness that resulted in a depth of 100 mm.

Conventional patient-specific IMRT QA in our institution consists of three verification procedures. Schematic layouts of our IMRT QA are shown in Fig. 1. Absolute point dose was measured using a PinPoint ionization chamber (model PTW31016; PTW, Freiburg, Germany), which was inserted into a verification phantom named I'm*RT* Phantom (IBA Dosimetry GmbH, Schwarzenbruck, Germany). The IMRT planned dose was recalculated at the I'm*RT* Phantom with a voxel size of 1.0 × 1.0 × 1.0 mm^3^, and then three measurement points were selected with reference to the AAPM Task Group 120 [6]. All beams of each IMRT plan were delivered to the I'm*RT* Phantom in the same way as in clinical treatments. The measured dose was compared with the mean dose at a virtual volume of the PinPoint ionization chamber in the TPS.

The composite planar dose distribution was measured using GAFCHROMIC EBT3 film (International Specialty Products, Wayne, NJ), which was inserted into the I'm*RT* Phantom in the axial plane. After 24 h from IMRT planned dose delivery, the irradiated radiochromic film was scanned by flatbed scanner (model GT-X970; Seiko Epson Corp., Nagano, Japan), and three-channel RGB data were acquired at 150 dpi [16]. The RGB value was subsequently converted to absolute dose using a dose–response curve, which was calibrated once every three months. Film analysis was conducted with Dkan2 software (Oras, Osaka, Japan) using a 5%(global)/3 mm criterion.

Per-field coronal planar dose was measured at a gantry angle of 0° by a MapCHECK device (Sun Nuclear Corp., Melbourne, FL, USA). The MapCHECK was placed on the treatment couch with Tough water (Kyoto Kagaku Co. Ltd, Kyoto, Japan) of 80-mm thickness that resulted in a depth of 100 mm. The source-to-detector plane distance was 1000 mm. The reproducibility for absolute dose measurement was checked using a 100 × 100 mm^2^ open field prior to the delivery of the IMRT planned dose, and thus daily variation of the MapCHECK device was controlled within ±0.3%. The measured planar dose was analyzed using accessory software version 5.02.01 included in the MapCHECK device, and the gamma passing rates were evaluated using a 3%(global)/3 mm criterion with a 10% lower-dose threshold. The degree of agreement between the MapCHECK and data calculated in the TPS was characterized using the passing rate of diode detectors failing to have gamma <1.

### Dose prediction in 3D

The detailed description of 3D delivered dose prediction using our novel algorithm can be found elsewhere [9]. A brief description of the procedure will be provided here.

Four individual pieces of data are required for delivered dose prediction from the per-field measurement: the Digital Imaging and Communications in Medicine – Radiation Therapy (DICOM RT) plan, the DICOM RT structure set, the DICOM RT dose for each treatment angle, and the 2D relative dose error. The DVH parameters were not compared for this study; therefore, only the contour of the I'm*RT* Phantom was exported as the DICOM RT structure set. Similarly, the recalculated dose at the I'm*RT* Phantom was exported with a voxel size of 3.0 × 3.0 × 3.0 mm^3^ due to limitations in data processing. The difference in dose voxel sizes will affect both the dose distribution and the monitor unit value for each port; therefore, the monitor unit of voxel size of 3.0 × 3.0 × 3.0 mm^3^ was set at the same value to that calculated with 1.0 × 1.0 × 1.0 mm^3^ in order to tally the prescribed dosage.

The 2D relative dose error was created using in-house software. The coronal planar dose on the I'm*RT* Phantom at a 100-mm depth was exported from the XiO TPS with the voxel size of 1.0 × 1.0 × 1.0 mm^3^ for the first step. Dosimetry was subsequently conducted using the MapCHECK device with Tough water of 80-mm thickness. The measured planar dose obtained at a gantry angle of 0° was then exported with the grid size of 5.0 × 5.0 mm^2^. After the dose comparisons between the measured and the calculated using in-house software, the 2D relative dose error for the correlated dose grid was created for the coronal plane.

**Fig. 2.**
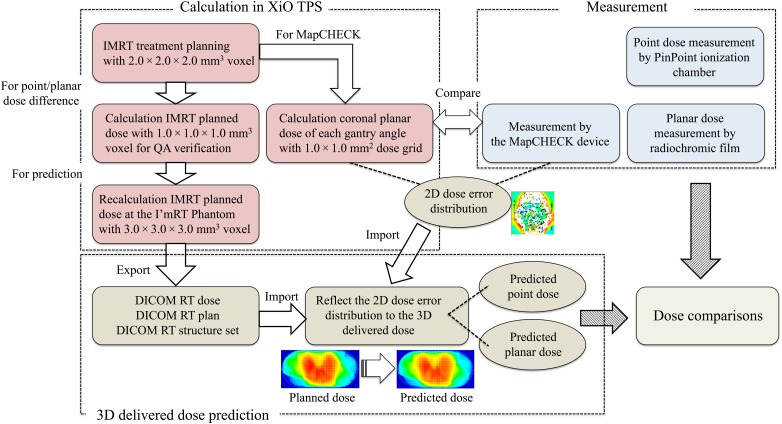
The flowchart of 3D delivered dose prediction. Four individual pieces of data are required for delivered dose prediction: DICOM RT plan, DICOM RT structure set, DICOM RT dose, and the 2D relative dose error in the coronal plane. The DICOM dataset was exported from the XiO TPS. The 2D relative dose error was created from dose comparisons between the measured and the calculated dose using in-house software.

The DICOM RT plan, DICOM RT structure set and the DICOM RT dose were imported to the prediction software for the first step. The original dose grid of each beam angle was read out but first transported into a gantry angle of 0°. The 2D relative dose error in the coronal plane was subsequently applied to the correlated dose grid. Finally, the error-reflected dose grid was re-transported to each original gantry angle. The summation of the error-reflected dose grid from each beam port allowed realistic prediction of the 3D delivered dose. A flowchart of the 3D delivered dose prediction is shown in Fig. 2.

### Criteria and statistical analyses

For the present study, comparisons of the measured dose, the dose calculated in the TPS, and the predicted dose were conducted. We retrospectively extracted both the measured and the calculated data for point dose difference and planar dose differences in the axial plane. The predicted 3D dose was reconstructed from the result of the per-field measurement, which was obtained in the same period as measurement of the point and planar doses. The criterion for point dose difference was set at ±3% tolerance for this study. Similarly, the criteria of the gamma index for planar dose differences were set at 3%(global)/3 mm, 4%(global)/3 mm and 5%(global)/3 mm so as to evaluate the sensitivity in the dose difference. Comparisons were made with the paired *t*-test or Wilcoxon signed rank test, depending on the normality of the samples, following the Shapiro–Wilk test. The two-sample Kolmogorov–Smirnov test was also conducted to determine whether the probability density of the two groups was equal or unequal. *P* < 0.05 was considered significant for the present study. All statistical analyses were performed using R version 3.1.2 statistical software (R Foundation, Vienna, Austria).

## RESULTS

**Fig. 3.**
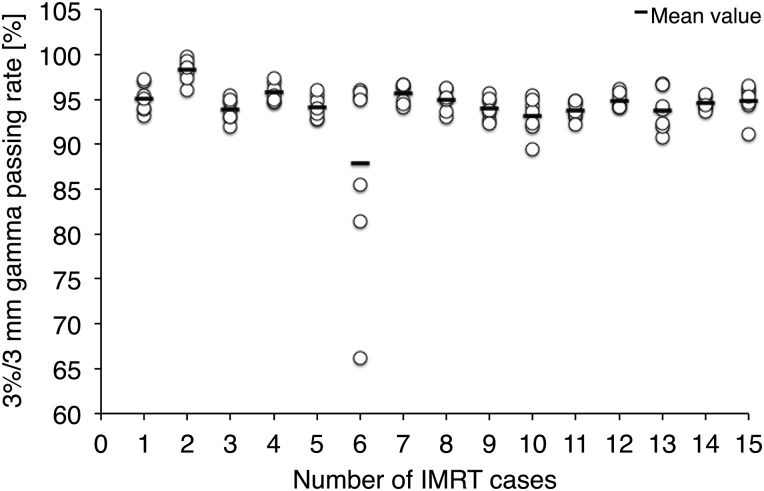
The results of gamma evaluation for the per-field measurement are shown. The horizontal axis shows the number of IMRT cases, and the vertical axis is the percentage gamma passing rate. Data were analyzed using accessory software included in MapCHECK device, and the dose differences were evaluated using the 3%(global)/3 mm criterion with a 10% lower dose threshold.

Figure 3 shows the gamma passing rates for the per-field measurement. The horizontal axis shows the number of IMRT cases, and the vertical axis indicates the gamma passing rate using a 3%(global)/3 mm criterion. A total of 96.2% of beam ports showed a >90% gamma passing rate. The lower gamma passing rates were observed for IMRT case number six and were due to the tongue-and-groove effect.

**Fig. 4.**
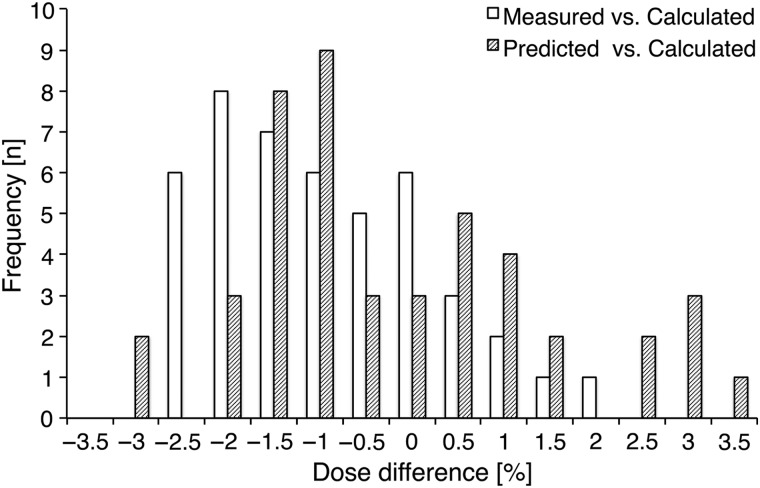
The histogram of point dose difference for ‘Measured vs Calculated’ and ‘Predicted vs Calculated’ is shown. The horizontal axis shows the percentage dose differences, and the vertical axis is the frequency. ‘Measured’ represented the dose measured by the PinPoint ionization chamber. ‘Calculated’ represented the dose calculated in the XiO TPS. ‘Predicted’ represented the 3D reconstructed dose using our prediction software with per-field measurement data alone.

Figure 4 shows the variations in point dose difference. The horizontal axis shows the percentage dose difference, and the vertical axis indicates the frequency. All differences between the measured and the calculated dose in the TPS were within ±3% tolerance, and the mean dose difference was −1.19% ± 1.16%. For the differences between the predicted and the calculated dose, 93.3% were also within ±3% tolerance, and the mean dose difference was −0.51% ± 2.38%. Three points exceeded ±3% tolerance between the predicted and the calculated dose; however, these dose differences were −3.11%, −3.05% and 3.04%. Similarly, the mean dose difference between the measured and the predicted dose was 0.15% ± 1.75%. Table 1 summarizes the *P*-values of both statistical analyses for the point dose difference. The two-sample Kolmogorov–Smirnov test for all combinations revealed no significant differences. However, the paired *t*-tests for ‘Measured vs Calculated’ and ‘Predicted vs Calculated’ revealed significant differences.

**Table 1.** The *P*-values for both statistical analyses (Kolmogorov–Smirnov test and paired *t*-test) of point dose difference are shown

	Significance of point dose difference	
	Kolmogorov–Smirnov test	Paired *t*-test
Measured vs Calculated	NS	*P* < 0.05
Predicted vs Calculated	NS	*P* < 0.05
Measured vs Predicted	NS	NS (*P* = 0.61)

*P* < 0.05 was considered significant for this study. NS = not significant.

**Fig. 5.**
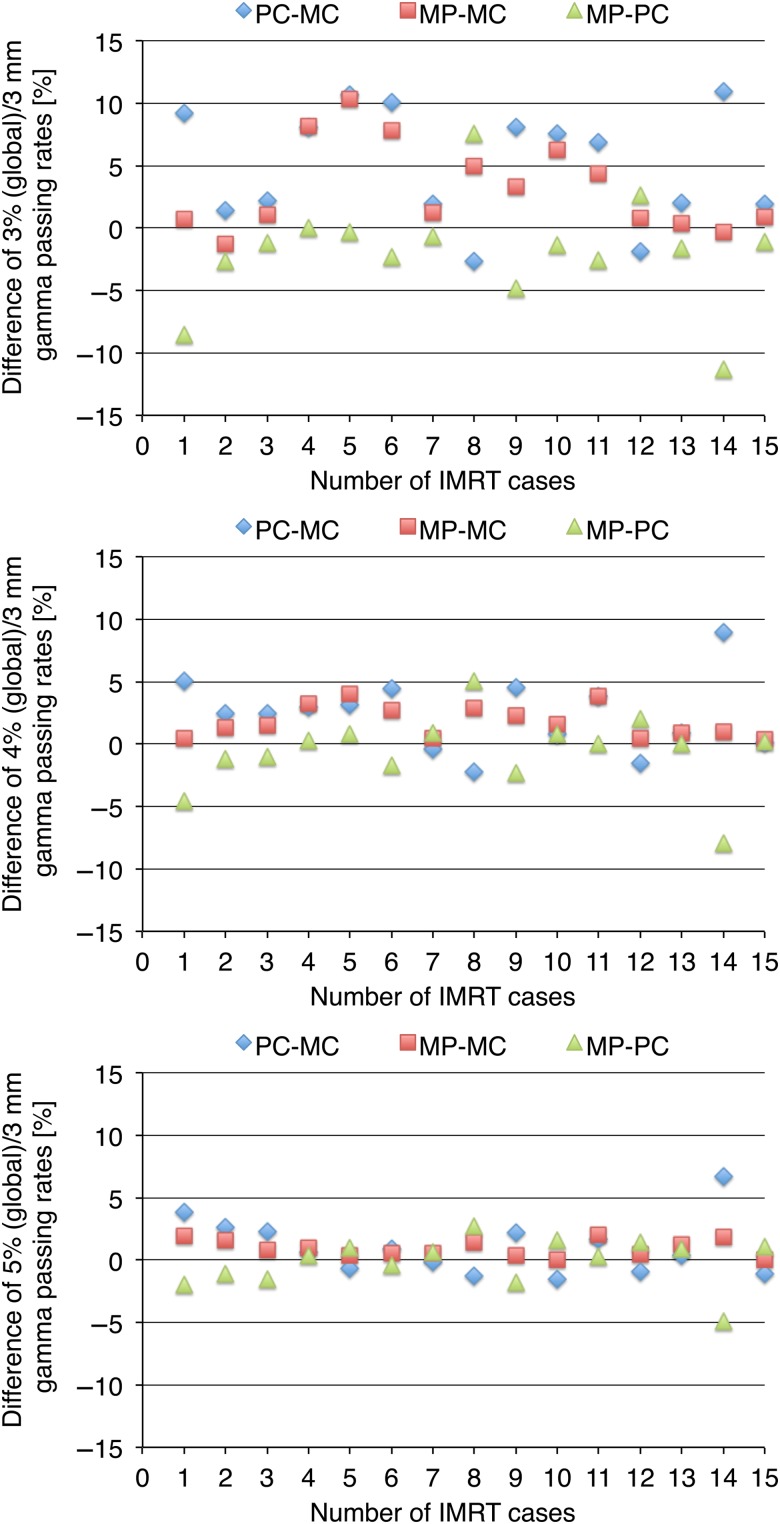
The differences in gamma passing rates for planar dose under the criteria of (a) 3%(global)/3 mm, (b) 4%(global)/3 mm and (c) 5%(global)/3 mm are shown. The horizontal axis shows the number of IMRT cases, and the vertical axis is the percentage gamma passing rate. MC = the gamma passing rate for ‘Measured vs Calculated’, PC = the gamma passing rate for ‘Predicted vs Calculated’, MP = the gamma passing rate for ‘Measured vs Predicted’.

Figure 5 shows the gamma passing rates for planar dose differences under the criteria of 3%(global)/3 mm, 4%(global)/3 mm and 5%(global)/3 mm. The horizontal axis shows the number of IMRT cases, and the vertical axis indicates the percentage gamma passing rate. The mean passing rates between the measured and the calculated doses were 85.0% ± 4.9% for 3%(global)/3 mm, 92.0% ± 3.0% for 4%(global)/3 mm and 95.8% ± 2.1% for 5%(global)/3 mm. Similarly, the mean passing rates between the predicted and the calculated doses were 90.1% ± 5.2%, 94.4% ± 3.7% and 96.9% ± 2.3%. Furthermore, the mean passing rates between the measured and the predicted doses were 88.2% ± 4.4%, 93.9% ± 3.3% and 96.8% ± 2.2%. Table 2 summarizes the *P*-values of both statistical analyses for planar dose difference. As with the results for point dose difference, the two-sample Kolmogorov–Smirnov test revealed no significant differences for any criteria. However, the paired *t*-tests for the combination of ‘Measured vs Calculated’ and ‘Measured vs Predicted’ doses revealed significant differences for all criteria. Similarly, significant differences were observed between ‘Measured vs Calculated’ and ‘Predicted vs Calculated’ doses for 3%(global)/3 mm and 4%(global)/3 mm criteria.

**Table 2.** The *P*-values for both statistical analyses of planar dose difference are shown

Criteria for gamma evaluation	Kolmogorov–Smirnov test			Paired *t*-test		
	MC vs PC	MC vs MP	PC vs MP	MC vs PC	MC vs MP	PC vs MP
3%/3 mm	NS	NS	NS	*P* < 0.01	*P* < 0.01	NS
4%/3 mm	NS	NS	NS	*P* < 0.05	*P* < 0.01	NS
5%/3 mm	NS	NS	NS	NS	*P* < 0.01	NS

The gamma passing rates of each combination were analyzed using the two-sample Kolmogorov–Smirnov test and the paired *t*-test. *P* < 0.05 was considered significant for this study. MC = the gamma passing rate for ‘Measured vs Calculated’, PC = the gamma passing rate for ‘Predicted vs Calculated’, MP = the gamma passing rate for ‘Measured vs Predicted’, NS = not significant.

## DISCUSSION

Conventional patient-specific IMRT QA requires several procedures for verifying that multiple fluctuating beam intensities can be delivered to the patient within the tolerance of each institution. Several dosimetric tools such as an ionization chamber, 2D diode array detectors and film are used for physical QA verifications; however, they place a significant workload on medical physicists. Pulliam *et al*. suggested that certain patient-specific QA procedures such as point and planar dose verifications could be curtailed because the gamma failing rates of verification tests were slightly low [8]. However, they indicated that errors would likely still occur, even though the frequency was low. The idea of curtailing certain QA procedures without any provision was, therefore, undesirable. In the present study, we proposed a novel QA curtailing strategy using the 3D dose prediction method.

For point dose difference, the variations between the measured and the calculated dose in the TPS were within ±3% tolerance. Furthermore, 93.3% of the point dose difference between the predicted and the calculated were also within ±3% tolerance. The predicted dose appeared to have good agreement with the measured dose, and there were no significant differences between the predicted and the measured doses with regard to either the two-sample Kolmogorov–Smirnov test or the paired *t*-test. Consequently, we concluded that the predicted dose was comparable with the dose measured by the PinPoint ionization chamber. The prediction-based approach, therefore, can be substituted for measurement in the verification of the point dose difference. Yin *et al*. noted that the field size affected the response of solid-state detectors due to limitation of the implemented theory [17]. Olch also confirmed the same effect from the small size of segments; hence, readout correction of the 1% under-reading effect of the diode array detectors should be conducted before dose comparisons [13]. For the present study, however, the mean dose difference between the measured and the predicted doses was 0.15% ± 1.75%, indicating that there was no need to correct the under-reading effect. The object of this study was IMRT QA for the pelvic region, in which a considerably large tumor is present; hence, the under-reading effect did not appear, owing to the large size of segments.

For the planar dose difference on the axial plane, a large number of diode detectors in the MapCHECK device were distributed in the major part of the PTV (because of the large size of the irradiation field in the case of IMRT for the pelvic region). By contrast, the number of detectors distributed around the peripheral region of the PTV, affecting the predicted distance-to-agreement, was considerably less; therefore, we evaluated the sensitivity in the variety of dose difference using three different criteria for the present study. The ‘Measured vs Predicted’ group obtained a higher mean gamma passing rate than the ‘Measured vs Calculated’ group for all criteria, and the differences were 3.2% for 3%(global)/3 mm, 1.9% for 4%(global)/3 mm and 1.0% for 5%(global)/3 mm. There were no significant differences between the ‘Measured vs Predicted’ and the ‘Measured vs Calculated’ doses for all criteria with regard to the two-sample Kolmogorov–Smirnov test; however, significant differences were observed with regard to the paired *t*-test. Since the 2D dose error distribution was applied to the predicted planar dose, it represented a more realistic dose distribution than that of the calculated dose distribution in the TPS. Furthermore, the 3D delivered dose prediction can provide a more precise verification for patient-specific IMRT QA because it includes uncertainty from MapCHECK measurements alone. By contrast, film dosimetry is affected by several uncertainties: the uniformities of scanner and films, the accuracy of the dose–response curve, and the precision of the film handling in dosimetry and analysis [18, 19]. Therefore, the prediction-based approach would be a robust technique compared with the measurement-based approach. For this reason, the ‘Predicted vs Calculated’ group obtained a higher mean gamma passing rate than the ‘Measured vs Calculated’ group for all criteria. Although the ‘Predicted vs Calculated’ group also obtained a higher mean gamma passing rate than the ‘Measured vs Predicted’ group for all criteria, no significant differences were observed. Therefore, we concluded that the predicted planar dose was comparable with the measured dose. The prediction-based approach can also be substituted for the measurement-based approach in the verification of planar dose differences.

Consequently, the novel prediction method could curtail the conventional IMRT QAs of point dose and planar dose that were performed using the ionization chamber and film. As described in the report of Van Esch *et al*. the conventional mean physicist's time typically needed per patient for point dose and planar dose verification distributed ranges from 6–40 h. In contrast, by applying the novel prediction method, we estimated the total time for IMRT QA per patient would be finished a maximum of 1 h, based on our experience. The extraction of 2D dose errors from MapCHECK dosimetry would be finished within 40 min, and the predicted point dose and planar dose would be reconstructed within 20 min.

Since the 2D relative dose errors were obtained from dosimetry at the gantry angle of 0°, the impact from actual gantry angle to the MLC movement was not reflected in the present evaluation. Our previous study reported that the accuracy of 3D dose prediction was significantly improved when the impact from actual gantry angle was taken into account in the prediction, although that study was conducted with a double-focusing MLC system [10]. However, for the present study, a single-focusing 160 MLC was used, which was independent of the impact of gantry angle. Our monthly non-gap test determined that there were no major differences in the gap intensity for every MLC abutment position between gantry angles of 0° and 180° (detailed data not shown). Therefore, the impact from the actual gantry angle was considered to be small for our single-focusing MLC system, but the institutions that use a double-focusing MLC system should take the effect of gantry angle into consideration for more precise prediction.

For the present study, the 3D delivered dose prediction was conducted with a voxel size of 3.0 × 3.0 × 3.0 mm^3^. However, the effect of a comparatively large voxel size would be small, both for point dose difference and planar dose differences. This is because the dose comparisons were performed inside a homogeneous phantom; furthermore, the dose gradient of the measurement location became precipitous from the grid-size recalculation. It is planned that the prediction technique will be improved to be able to handle a smaller size dose grid in order to extend the applicable range of our software to a smaller tumor.

The gamma passing rate of IMRT case number six was quite small with regard to the per-field measurement. We analyzed where the dose errors (such from tongue-and-groove effect) occurred inside the PTV and concluded that they had a small effect on the treatment. As a consequence, the gamma passing rate for the planar dose differences of case six was comparable with that of the other IMRT cases without modifications. Kruse reported that a per-field measurement at a gantry angle of 0° was not sensitive to dosimetric inaccuracies in the IMRT plans [4]. This fact suggested that there was no correlation between the 2D gamma passing rate obtained by per-field measurements and the 3D composite gamma passing rate. Filtering by gamma passing rate of the per-field measurement, therefore, should not be performed for the 3D delivered dose prediction because the objective of 2D measurements was merely to extract the dose error distribution of each beam port.

The important point is to comprehend how 2D error distribution will affect patient DVHs. Stasis *et al*. reported weak correlations between the gamma passing rates of per-field measurements and the patient DVHs, indicating a limitation for application of physical QA indications [20]. As a novel evaluation approach, Zhen *et al*. incorporated biomathematical treatment outcome models of tumor control probability (TCP) and normal tissue complication probability (NTCP) into the patient-specific IMRT QA [21]. They suggested that the TCP/NTCP models are effective tools for integration of physical QA indications into biological evaluation. The implementation of these models into the prediction software will allow us to evaluate how the physical error distribution would affect the therapeutic goal or the side effects occurring from the per-field measurement alone.

## CONCLUSION

Current patient-specific IMRT QA may have procedures that reduce cost effectiveness, even though the IMRT plans must be validated to confirm that they are able to deliver the planned dose successfully. From the comparisons between the measured, calculated and predicted data with regard to point dose and planar dose differences, the predicted dose was comparable with the measured dose. We concluded that the prediction-based approach can be substituted for the conventional measurement-based approach without any constraint. This novel approach will help medical physicists save time on IMRT QA for each patient.

## FUNDING

Funding to pay the Open Access publication charges for this article was provided by a grant from the Japan Society for the Promotion of Science (JSPS) Core-to-Core Program (Grant No. 23003).
